# Double Guided Surgery in All-on-4^®^ Concept: When Ostectomy Is Needed

**DOI:** 10.1155/2018/2672549

**Published:** 2018-02-04

**Authors:** Gabriele Tonellini, Raquel Saez Vigo, Giorgio Novelli

**Affiliations:** San Gerardo Hospital, Department of Maxillofacial Surgery, University of Milano-Bicocca, Milan, Italy

## Abstract

**Background:**

The rehabilitation of edentulous jaws with guided and flapless surgery applied to the All-on-4 concepts is a predictable treatment with a high implant and prosthetic survival rates, but there are several contraindications for this technique like when bone reduction is needed due to a high smile line in the maxilla or when there is an irregular or thin bone crest.

**Purpose:**

To report a technique with double guided surgery for bone reduction and implant placement with the All-on-4 concept.

**Materials and Methods:**

7 patients were included in the study. Guided implant planning was performed using CBCT, and the virtual templates were created with three dedicated software. Custom surgical templates were made for the ostectomy and for implants positioning.

**Results:**

28 implants were placed using a double bone-supported surgical guide. The mean angular errors between the preoperative-planned implant and the postoperative-placed implant were 2.155° ± 2.03°; the mean distance errors between the planned and the placed implants were 0.763 mm ± 0.55 mm on the shoulder implant and 0.570 mm ± 0.40 mm on the apex implant.

**Conclusions:**

The results of our study indicate that this treatment is predictable with an excellent survival rate allowing excellent results even when bone reduction is mandatory.

## 1. Introduction

One of most important things for edentulous rehabilitation is to optimize the patient's treatment and comfort in the fastest and safest way. In the last years, the use of one-stage surgical protocols with immediate function has demonstrated to be an effective treatment in full or partial-arch edentulous rehabilitation, giving patients the chance of having a fixed dentition as soon as possible [1].

Sometimes, the lost of posterior teeth of the mandible can make complex the treatment plan because of the impediment in using the alveolar bone posterior to the inferior alveolar nerve without the addition of complicate surgical steps like bone grafting procedures or nerve transposition [2].

The same can happen in the maxilla when the atrophic bone makes difficult the rehabilitation without a sinus lift.

The All-on-4 treatment concept introduced by Maló allows the rehabilitation of edentulous jaws without bone graft in one surgical step through the placement of 4 implants, optimizing the available bone. The implants are placed two posteriorly tilted between 30° and 45° and two anteriorly axial, well anchorated achieving a primary stability of at least 30 Ncm. The survival rate implant related was 98% for the maxilla and 98.1% for the mandible after 5 to 10 years of follow-up [3–5]. The use of tilted and longer implants increases primary stability, allows cantilever decrease with excellent prosthetic support, and maximizes the use of available bone [6].

The clinical outcome of optimal implant placement is based on precise preoperative planning. Computer-aided surgery techniques are suggested for reaching a precise implant position avoiding lesions for important anatomical structures such as the maxillary sinus or the mandibular nerve [7].

Several authors introduce a variance from the protocol presented by Maló using the guided surgery for the All-on-4 procedure [8, 9]. According to the guided surgery protocol, a surgical guide is made based on data obtained through CBCT [10].

Naziri et al. have proven the precision and predictability of implant placement when using CAD-CAM surgical guides based on CBCT. The analysis of Naziri shows a median deviation between preoperative plan and postoperative implant positions of 1 mm at the implant shoulder and 1.4 mm at the implant apex, with a median angular deviation of 3.6° [11].

There are three kinds of tissue that can sustain stereolithographic surgical guides: bone, mucosa, and tooth. The first templates used for the treatment of edentulous full-arch were the bone-supported guides. In 2006, Fortin et al. introduced the flapless surgical technique with mucosa- and tooth-supported template. This is a minimally invasive technique that allows us to decrease surgical time, patient discomfort, postoperative bleeding, and the healing period, but it is important to remember that bone template provides the best visualization of the surgical field, allowing for better control of the whole procedure [7, 12, 13].

The results of Maló studies suggest that the rehabilitation of edentulous jaws using surgical planning and surgical-customized templates with prosthetic rehabilitation through CBCT, CAD-CAM technology, and flapless surgery is a predictable treatment with a high implant and prosthetic survival rates when is applied to the All-on-4 concept. However, there are several contraindications for this technique; one of the most important is when bone reduction is necessary due to a gummy smile in the maxilla or when an irregular or thin bone crest in the jaws prevents a correct treatment [6].

The smile line must be considered when planning an implant-supported fixed prosthesis. We must ensure that the prosthesis tissue junction (PTJ) is not visible during the patient's maximum smile. This is primarily because of the difficulty to match with precision of the colour of the prosthetic gingiva with the natural gingival tissues [14–17].

The second cause for bone reduction is to allow adequate implant and prosthetic space. In all these cases, it is necessary to perform a bone reduction or ostectomy of the jaws, but it is no easy to know how many bone it must be reduced; underreduction of bone can lead to prosthetic failure, and overreduction of bone can produce a divestment of available bone and risks encroachment of vital anatomic structures [14–16].

This article describes a technique with double-guided surgery for bone reduction and implant placement in the All-on-4 concept, avoiding risks of vital anatomic structures and guarantying a good aesthetic result. This protocol can be used with edentulous patients and also patients with failure dentition.

## 2. Materials and Methods

Seven patients with edentulous or partial edentulous arches were included and treated in 3 private center practices. The patients were 45 to 72 years old.

A total of 28 implants were placed between February 2015 and October 2016.

The treatment's plannings were performed always by the same surgeon.

Four implants were placed in the maxilla and 24 implants in the mandible. One ostectomy guide was used in the maxilla, and 6 were used in the mandible.

The procedure and the evaluation of the aesthetic parameters were based on a planning data and 2D photographs. A prosthesis was manufactured prior to the implant surgery and was immediately inserted after surgery.

Panoramic radiographs and CT scan were examined.

Patients with minimum bone volume available with thin crest bone or with gingival display to perform an All-on-4 rehabilitation were selected, so patients with bone reduction were nedeed.

### 2.1. Planning Protocol

Guided implant planning was performed using CBCT, and computer-assisted implant treatment planning software 3Diagnosys (3Diemme, Cantú, Italy), Mimics 10.01 (Materialise, Leuven, Belgium), and PlastyCAD 1.5 (3Diemme) were used to create the virtual templates.

**Figure 1:**
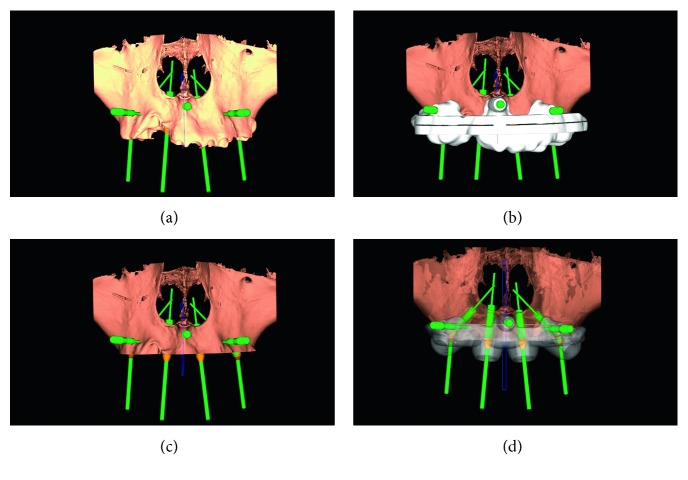
(a) 3D implant planning. (b) Resection guide. (c) 3D implant planning with ostectomy performed. (d) Implant guide.

Custom surgical templates were made for the ostectomy and for implants position (3Diemme, Cantù, Italy) (Figure 1).

The planning protocol includes alveolar ostectomy of the maxilla up to 2 mm from line smile when there was a gingival display and as much as necessary bone reduction when there was an irregular or thin crest in the maxilla or in the mandible. The measurements were made directly on the patient and then reported to the software.

The implants were planned according to the All-on-4 protocol, two tilted and two axial, to take advantage of the available bone. The implants were not prosthetically driven.

The STL file of templates was then sent to fabricate. These templates were made in all-acrylic resin with 3D DWS Digitalwax 020D printer that could print with a minimum of 0.01 mm thickness.

### 2.2. Surgical Protocol

The surgical procedures for both jaws were performed under local anaesthesia with sedation. Antibiotics (clavulanic acid + amoxicillin) were given 1 hour before surgery and daily for six days thereafter. Prednisone was administrated daily in a regression mode (from 15 mg to 5 mg) from the day of surgery until 4 days postoperatively. Analgesics were given for 4 days and then just if needed.

A mucosal incision was made to raise a mucoperiosteal flap; the bone-supported surgical template for ostectomy was positioned and fixed with three anchor pins. Then the ostectomy was performed with a saw (W&H).

After the ostectomy, the second template was fixed in the same holes of the first anchor pins. The precise fit of surgical templates was visually and manually checked before surgery.

**Figure 2:**
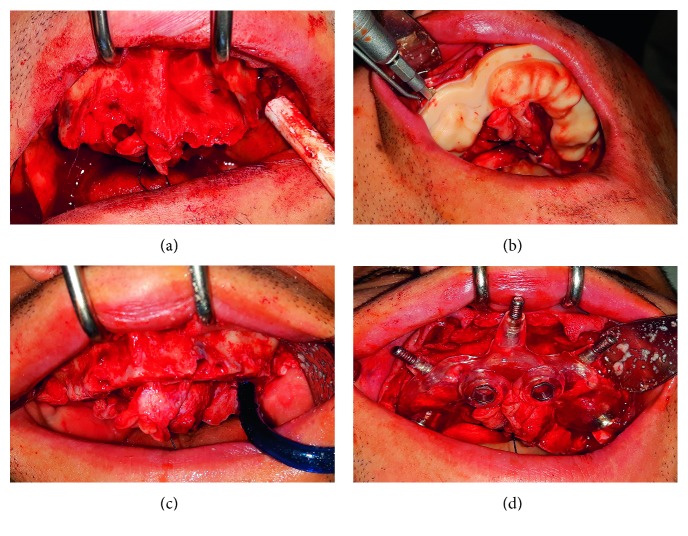
(a) Maxillary postextraction. (b) Ostectomy performed by saw through the guide. (c) Removal of the ostectomized bone. (d) Implant guide placed on the same holes used to fix the resection guide.

Implants were placed through the sleeves of the surgical template in the planning anatomic sites (Figure 2). Four different types of implants were used, *Nobel speedy*, *Nobel parallel CC*, *Prodent twinner collar*, and *Leader Implus*, depending on the preference of implant connection required by the dentist. The implant site was under preparation according to the bone density achieving an insertion torque of 35 to 50 Nmc in the maxilla, and 30 to 70 Nmc in the mandible was applied to obtain a primary stability for loading immediately the fixed denture prosthesis.

### 2.3. Immediate Provisional Prosthetic Protocol

Implant-supported fixed prosthesis of high-density acrylic resin with titanium cylinders were manufactured at the dental laboratory and inserted at the same day. The provisional prosthesis was positioned in the mouth using the patient's occlusion. Just anterior occlusal contacts were preferred in the provisional prosthesis, and no cantilevers were used. Emergence positions at the posterior implants were normally at the second premolar or first molar allowing the prosthesis to hold 10 to 12 teeth [18].

### 2.4. Outcome Measures

**Figure 3:**
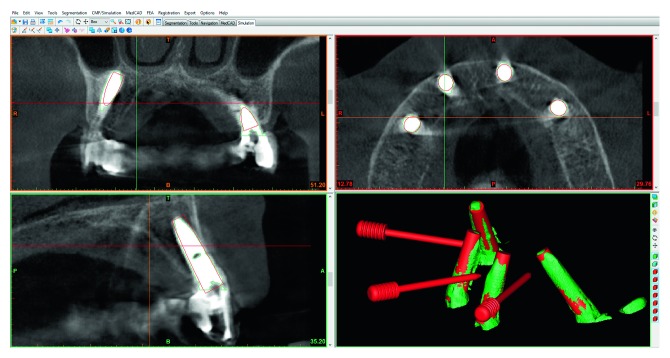
Super imposition of the postop CT scan and the preop 3D panning.

The first parameter evaluated was the accuracy of implants position according to the surgical planning. It was confronted that the 3D CT scan reconstruction of the planning is obtained with 3Diagnosys software with a postsurgery CT scan. In order to analyse differences between preoperative planned implants and postoperative placed implants, angular errors and distance errors were evaluated [19] (Figure 3).

The second outcome evaluated was the implant survival rates. To analyse this parameter, Maló Clinic survival criteria were used: clinical stability, function without any discomfort, absence of suppuration, infection, or radiolucent areas around the implants during the follow-up [6].

**Figure 4:**
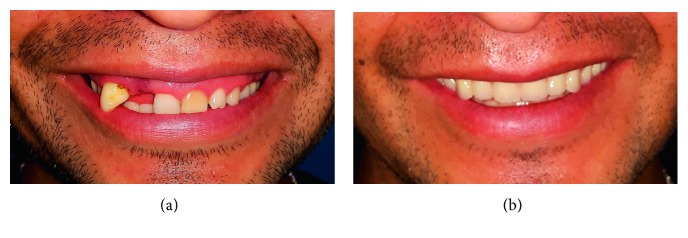
(a) Preop clinical presentation. Partially edentulous with high mobility of the teeth and gummy smile. (b) Immediate postop with provisional prosthesis. With ostectomy of the maxilla we correct the defect of the patient's smile.

The third and last outcome evaluated was the aesthetic of smile with the fixed complete denture prosthesis. “Dental aesthetics” has been defined as “the application of the principles of esthetics to the natural or artificial teeth and restorations.” It is difficult to find studies in the literature that can be considered as evidence based. The parameters considered in this study were the concealment of prosthesis tissue junction and an adequate posterior tooth extension to avoid “black space” behind the prosthesis [15, 16] (Figure 4).

## 3. Results

Twenty-eight implants were placed in 7 patients, and all implants were inserted using bone-supported surgical guide, created with 3 dedicated software. To place the implants, the All-on-4 protocol was performed. All implants were loaded immediately.

The angular and distance errors are summarized in Table 1. The mean angular error between the preoperative planned implant and the postoperative placed implant was 2.155 ± 2.03; the mean distance errors between the planned and the placed implants were 0.763 ± 0.55 on the shoulder implant and 0.570 ± 0.40 on the apex implant.

Life table analysis is reported in Table 2. At one year of follow-up, 0 implants failed, resulting in a cumulative implant survival rate of 100%; all implants are functional with 0% of infection or radiolucent areas. It was not reported any complication during the entire follow-up.

**Figure 5:**
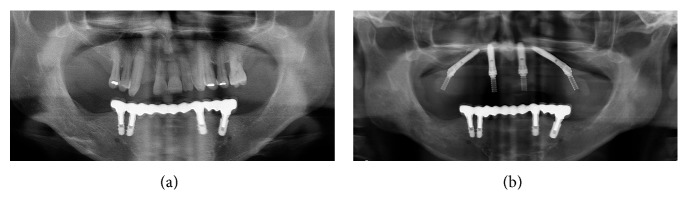
Pre- and post-OPG. In the postop OPG, we can see the guided ostectomy of the maxilla and the implants positioning.

In all patients, the prosthesis tissue junction was not visible during the maximum smile, and there was no black space posterior on prosthesis. The aesthetic result evaluated from patients was excellent (Figure 5). The aesthetic parameters results are summarized in Table 3.

## 4. Discussion

The present study was planned to estimate the accuracy of implants position, the survival rate of implants placed, and the aesthetic of smile using a new protocol that expects a double surgical guide. The first guide is to perform an ostectomy in all cases when bone reduction is mandatory, and the second guide is to place the implants in a perfect position to avoid anatomical damages and avoiding bone grafts. The protocol was planned first virtually with computer-assisted planning software, and the virtual templates with bone support were created. Computer-guided implant surgery consents accurate implant positioning with safety application and has the advantage of surgical time reduction and the optimization of available bone [20]. The templates used were bone supported as we must raise the flap to perform the bone reduction; this kind of templates provides better accuracy than conventional flapless guides because the limiting factor of soft tissue is removed after flap elevation [7]. The fit of template was based on bone anatomy, and the soft tissue does not interfere with it. Our technique offers the option to fabricate a guide with increased thickness that improves the mechanical proprieties avoiding the fracture of the guide during the surgery.

With this method it is not necessary to use the classic protocol of double CT scanning, like flapless guided surgery, to obtain the gingival surface because we use only bone surface to create the template. So the time and the final cost of the treatment were reduced. Another advantage over the free-hand approach is the precision of implant positioning using all distal available bone and the accuracy of the millimeters of ostectomy.

The results of this study are in agreement with previously published works [20–25].

The transfer of the virtual planning to the surgical template for the operation time results in a very accurate technique.

Nowadays, it is mandatory to expect the best aesthetic results so as to correct the excessive gingival display which becomes also a priority, such as to prevent black spaces posterior in the final prosthesis and a good outcome to the PTJ. It does not exist as a simple technique to perform bone reduction with a guided surgery in a safest and quick way [26–28].

## 5. Conclusions

The results of this preliminary study suggest that this treatment modality for total or partial edentulous patients is predictable with an excellent implant survival rate. By combining 3D planning for a double surgical guide, the All-on-4 protocol, and immediate loading implants, it is possible to increase the advantages of each one, resulting in a more accurate and safer technique with high predictable results. Patients can rehabilitate full-arches even when bone reduction is mandatory because of a gummy simile or because of an irregular or thin bone crest. Our technique demonstrated excellent aesthetic outcomes with a reduction surgery time without any complication.

## Figures and Tables

**Table 1:** Accuracy: angular and distance errors.

Patient	Position of implant	Angular error (°)	Shoulder error (mm)	Apical error (mm)
1	#45	0.65	0.1	0
	#42	0	0.1	0.5
	#32	3.37	1.37	1.09
	#35	4.19	0.91	0.72
2	#15	0.67	0.59	0.75
	#12	3.40	0.45	0.66
	#22	4.11	1.33	0.82
	#25	0.83	1.77	0.20
3	#45	0	0.75	0.1
	#42	2.21	0.47	0.42
	#32	9.49	0.36	1.82
	#35	2.99	0.40	0.89
4	#45	5.56	1.22	0.27
	#42	1.09	0.39	0 58
	#32	0	0	0
	#35	0.79	0.80	0.42
5	#45	2.03	1.01	0.32
	#42	1.01	0.30	0.65
	#32	3	0.81	0.87
	#35	1.05	0.93	0.41
6	#45	0.80	0.23	0.29
	#42	2.03	1.76	1.05
	#32	1.10	1.14	0.58
	#35	3.01	0.89	0.27
7	#45	3.21	1.79	1.20
	#42	0.65	0.40	0.46
	#32	2.20	0.95	0.62
	#35	0.9	0.32	0.31
Mean ± standard deviation	—	2.155 ± 2.03	0.763 ± 0.55	0.570 ± 0.40

**Table 2:** Implant survival rate.

	Patient 1	Patient 2	Patient 3	Patient 4	Patient 5	Patient 6	Patient 7
Number of implants stable	4	4	4	4	4	4	4
Number of implants functional	4	4	4	4	4	4	4
Number of implants with infection	0	0	0	0	0	0	0
Number of implants with radiolucent areas	0	0	0	0	0	0	0

**Table 3:** Aesthetic parameter results.

	Patient 1	Patient 2	Patient 3	Patient 4	Patient 5	Patient 6	Patient 7
PTJ visible	No	No	No	No	No	No	No
Black space posterior	No	No	No	No	No	No	No
